# 5mC regulator-mediated molecular subtypes depict the hallmarks of the tumor microenvironment and guide precision medicine in bladder cancer

**DOI:** 10.1186/s12916-021-02163-6

**Published:** 2021-11-26

**Authors:** Jiao Hu, Belaydi Othmane, Anze Yu, Huihuang Li, Zhiyong Cai, Xu Chen, Wenbiao Ren, Jinbo Chen, Xiongbing Zu

**Affiliations:** 1 Department of Urology, Xiangya Hospital, Central South University, Changsha, 410008 China; 2 Immunobiology & Transplant Science Center, Houston Methodist Research Institute, Texas Medical Center, Houston, TX 77030 USA; 3 Institute for Infection Prevention and Hospital Epidemiology, Medical Center, University of Freiburg, Freiburg, Germany; 4 George Whipple Lab for Cancer Research, Departments of Pathology and Urology, and The Wilmot Cancer Institute, University of Rochester Medical Center, Rochester, NY 14646 USA

**Keywords:** Bladder cancer, 5-Methylcytosine, Molecular subtype, Tumor microenvironment, Immune phenotype, Immunotherapy

## Abstract

**Background:**

Depicting the heterogeneity and functional characteristics of the tumor microenvironment (TME) is necessary to achieve precision medicine for bladder cancer (BLCA). Although classical molecular subtypes effectively reflect TME heterogeneity and characteristics, their clinical application is limited by several issues.

**Methods:**

In this study, we integrated the Xiangya cohort and multiple external BLCA cohorts to develop a novel 5-methylcytosine (5mC) regulator-mediated molecular subtype system and a corresponding quantitative indicator, the 5mC score. Unsupervised clustering was performed to identify novel 5mC regulator-mediated molecular subtypes. The principal component analysis was applied to calculate the 5mC score. Then, we correlated the 5mC clusters (5mC score) with classical molecular subtypes, immunophenotypes, clinical outcomes, and therapeutic opportunities in BLCA. Finally, we performed pancancer analyses on the 5mC score.

**Results:**

Two 5mC clusters, including 5mC cluster 1 and cluster 2, were identified. These novel 5mC clusters (5mC score) could accurately predict classical molecular subtypes, immunophenotypes, prognosis, and therapeutic opportunities of BLCA. 5mC cluster 1 (high 5mC score) indicated a luminal subtype and noninflamed phenotype, characterized by lower anticancer immunity but better prognosis. Moreover, 5mC cluster 1 (high 5mC score) predicted low sensitivity to cancer immunotherapy, neoadjuvant chemotherapy, and radiotherapy, but high sensitivity to antiangiogenic therapy and targeted therapies, such as blocking the β-catenin, FGFR3, and PPAR-γ pathways.

**Conclusions:**

The novel 5mC regulator-based subtype system reflects many aspects of BLCA biology and provides new insights into precision medicine in BLCA. Furthermore, the 5mC score may be a generalizable predictor of immunotherapy response and prognosis in pancancers.

**Supplementary Information:**

The online version contains supplementary material available at 10.1186/s12916-021-02163-6.

## Background

Bladder cancer (BLCA) is one of the most common urinary malignancies, and the tumor microenvironment of BLCA is significantly heterogeneous [1–4]. The prognosis of advanced BLCA is poor, despite promising progress in immune checkpoint blockade (ICB), neoadjuvant chemotherapy, and targeted therapy [5]. This is because a majority of BLCA patients are not sensitive to these therapies, and there are no robust and efficient biomarkers or tools to accurately predict the clinical response to those treatments. Therefore, developing reliable treatment response stratification biomarkers for achieving precision medicine is still challenging.

The tumor microenvironment (TME) is a heterogeneous system consisting of cancer cells, immune cells, and extracellular matrix [3, 4]. High TME heterogeneity reflects significantly distinct functional hallmarks in patients with the same pathological stage and grade, which may result in different clinical responses to the same treatments [6, 7]. Notably, high TME heterogeneity impedes the achievement of precision medicine in BLCA. Therefore, depicting TME heterogeneity could reveal many aspects of bladder cancer biology and further our insights into bladder cancer treatment. Developing novel therapeutic response predictors and therapeutic targets in the context of TME heterogeneity may be a promising path for achieving precision medicine in BLCA.

Molecular subtypes hold great promise in addressing TME heterogeneity and in precision medicine for BLCA [3]. Several molecular subtype systems have been developed based on RNA sequence data, such as the CIT, Lund, MDA, TCGA, Baylor, and UNC systems [8–14]. However, the clinical application of these molecular subtypes may be impeded by several issues, such as the complex RNA sequencing method, high economic burden, long detection period, and the nonnegligible diversity of these molecular subtype classifications. Therefore, more economical, rapid, and accurate molecular classification is required to promote precision medicine in BLCA. 5-Methylcytosine (5mC) in DNA, the most critical epigenetic modification, shapes TME heterogeneity by influencing genomic stability, determining the cancer cell differentiation state, and selecting cell identity [15–18]. In BLCA, DNA methylation plays critical roles in early diagnosis, predicting prognosis, predicting therapeutic opportunities, and acting as a potential therapeutic target [16, 19, 20]. Robertson et al. identified five DNA methylation-based subtypes that correlated with different biological characteristics, clinicopathological characteristics, and clinical outcomes based on cancer-specific hyper- or hypomethylated CpG sites in BLCA [8]. This evidence indicated that DNA methylation-based molecular subtypes could reflect the TME heterogeneity of BLCA. Nonetheless, the complex detection method of DNA methylation profiles and high economic burden limit the clinical application of DNA methylation-based molecular subtypes.

Global DNA methylation profiles depend on the cross-talk between three kinds of 5mC regulators: writers, erasers, and readers [21–24]. To date, the role of these 5mC regulators in shaping TME heterogeneity remains to be further investigated in BLCA. Thus, we take a global view of the mRNA expression levels of these 5mC regulators to assess their comprehensive relevance to TME heterogeneity, immune phenotypes, clinicopathological characteristics, and therapeutic opportunities for BLCA. Then, a novel 5mC regulator-mediated molecular subtype system was developed, and a 5mC score was generated to quantify these subtypes in BLCA.

## Methods

### Data retrieval and preprocessing

#### Xiangya cohort

Sixty fresh bladder cancer tissues and 13 paired normal bladder tissues were collected from our hospital and immediately stored in liquid nitrogen. We first extracted total RNA from fresh tissues using TRIzol (Invitrogen, Carlsbad, CA, USA). Subsequently, we quantified the total RNA using a NanoDrop and Agilent 2100 bioanalyzer (Thermo Fisher Scientific, MA, USA). After we constructed the mRNA library, we further purified and fragmented the total RNA into small pieces. Finally, we synthesized first-strand cDNA and second-strand cDNA, which were further amplified by PCR to construct the final library (single-stranded circular DNA). Eventually, 57 BLCA samples and 13 normal tissues were qualified and sequenced on a BGISEQ-500 platform (BGI-Shenzhen, China). The RNA sequencing data of these samples were analyzed with TPM values.

##### Single-cell RNA sequencing

One radical cystectomy sample of a patient (muscle-invasive bladder cancer, high grade) was collected from our hospital, and then it was run by single-cell RNA sequencing in OE Biotech Co, Ltd (Shanghai, China) (Xiangya scRNA set). The detailed sequencing procedures, data preprocessing, and analysis methods have been reported in previous studies [25, 26]. Briefly, the tumor samples were prepared into single-cell suspension, which was subsequently loaded on a Chromium Single Cell Controller instrument (10× Genomics, Pleasanton, CA, USA) to generate the single-cell gel beads in emulsions. The Cell Ranger (version 2.2.0) was used to process the raw data. The InferCNV package was used to detect the CNVs in all cells and to recognize real bladder cancer cells. Then, we explored the expression profiles of 5mC regulators on every cell. Also, we downloaded a public BLCA single-cell data set (GSE145137), which provided the cell cluster and cell type information [27]. Therefore, we directly visualized the expression patterns of 5mC regulators by using the “VlnPlot” function in “Seurat” package for this data set.

#### The Cancer Genome Atlas (TCGA) cohort

For BLCA, the R package TCGAbiolinks was used to download the RNA sequencing data (FPKM values), mutation profiles, and clinical data from the Genomic Data Commons (GDC, https://portal.gdc.cancer.gov/). Then, the FPKM values were transformed into transcripts per kilobase million (TPM) value. After filtering the genomic and clinicopathological data, a total of 400 BLCA samples were included in this study. Among these patients, 396 patients were diagnosed with muscle-invasive bladder cancer (MIBC), while the other four patients were diagnosed with non-MIBC (NMIBC). The copy number variation (CNV) data were gathered from the UCSC Xena data portal (http://xena.ucsc.edu/). The maftools R package was used to plot the somatic mutation data. For pancancers, the RNA sequencing data, mutation data, and survival information were downloaded from the UCSC Xena data portal. VarScan2 was used to analyze the mutation data and then calculate the tumor mutation burden (TMB). The microsatellite instability (MSI) data were collected from the supplementary files of Bonneville’s study [28]. The stemness indices of pancancers were gathered from Malta’s study [29].

#### Four external BLCA validation cohorts

Two BLCA cohorts with detailed survival data and the same sequencing platform, namely, GSE48075 and GSE32894, were downloaded from Gene Expression Omnibus (GEO). Then, we combined these two cohorts into a meta-cohort using the sva R package. Another cohort (E-MTAB-4321) with 476 BLCA samples was downloaded from the European Molecular Biology Laboratory database. In addition, the IMvigor210 cohort, which included 348 BLCA samples that received anti-PD-L1 immunotherapy, was obtained from http://research-pub.Gene.com/imvigor210corebiologies/.

#### Nine external immunotherapy cohorts

First, three immunotherapy cohorts were downloaded from the GEO database, including GSE135222 (non-small-cell lung cancer), GSE78220 (melanoma), and GSE91061 (melanoma). Then, another six immunotherapy cohorts with a detailed RNA expression matrix and clinical information were gathered from the TIDE website [30].

Detailed information about these cohorts is summarized in Table S1A, B, C, D, E, F, G.

### Unsupervised clustering of 21 5mC regulators

We systematically included 21 5mC regulators in this analysis from previous studies [21–24]. These 5mC regulators included 3 writers (DNMT3A, DNMT3B, and DNMT1), 4 erasers (TET1, TET2, TET3, and TDG), and 14 readers (MBD1, MBD2, MBD3, MBD4, MECP2, NEIL1, NTHL1, SMUG1, UHRF1, UHRF2, and UNG). Based on the expression profiles of these 5mC regulators in the training set (TCGA-BLCA cohort), the ConsensuClusterPlus R package was applied to perform unsupervised clustering analysis and then to identify distinct 5mC regulator-mediated molecular clusters comprehensively [31]. For this cluster algorithm, we selected the following parameters: 80% item resampling (pItem), 80% gene resampling (pFeature), a maximum evaluated *k* of 6 (maxK), 1000 resamplings (reps), and kmdist clustering algorithm (clusterAlg) upon 1-Spearman correlation distances (distance). Similarly, we also explored the purity corrected 5mC clusters. The tumor purity data of TCGA-BLCA was collected from the supplementary files of Aran’s study (Table S1H) [32]. There were five types of tumor purity data, including Estimate, LUMP, Absolute, IHC, and CPE. Consistent with the previous study, the CPE purity was used to adjust the original mRNA expression matrix [32].

### Identifying differentially expressed genes (DEGs) between 5mC clusters

We identified the DEGs between different 5mC clusters by using the empirical Bayesian approach of the limma R package. The significance criteria for determining DEGs were set as adjusted *P* value < 0.001 and |logFC| > 1.5. To investigate the functions of these DEGs, we conducted Gene Ontology (GO) and Kyoto Encyclopedia of Genes and Genomes (KEGG) analyses using the Metascape data platform [33]. These DEGs were called the 5mC gene signature in this study.

### Identifying differential methylation probes (DMPs) and developing DMP clusters

The level 3 methylation data of TCGA-BLCA (HumanMethylation450 platform) was gathered from the UCSC Xena data portal (http://xena.ucsc.edu/). The DNA methylation status for each methylation site (CpG) was evaluated by a beta (*β*) value, with scores of “0” representing no DNA methylation and scores of “1” representing complete DNA methylation. The ChAMP R packages and several related functions were used to process and analyze the DNA methylation data [34]. First, we screened the DMPs between bladder cancer and normal samples using the significance criteria of adjusted *P* value < 0.01. Furthermore, CpG sites that were not methylated in normal tissues (mean *β* value < 0.2) but were highly methylated in cancer tissues (mean *β* value > 0.5) were defined as the cancer-specific hypermethylated DMPs. In contrast, cancer-specific hypomethylated DMPs were defined as those DMPs which were not methylated in cancer tissues (mean *β* value < 0.2) but were highly methylated in normal tissues (mean *β* value > 0.5) [8, 35]. Also, we performed unsupervised clustering based on those cancer-specific hypermethylated and hypomethylated DMPs to identify DMP clusters. Similarly, we screened the significant DMPs between 5mC clusters (adjusted *P* value < 0.01); the same filter criteria determined the 5mC cluster-specific DMPs. We further identified the differential methylation genes (DMGs) based on these DMPs. The GO and KEGG pathway analysis of these DMGs were performed by using the Metascape data platform. Finally, we correlated the 5mC score with the promoter methylation status of certain critical cancer-associated genes, including oncogenes, tumor suppressor genes, driver genes, and kinase genes. We defined the 5′UTR, TSS1500, TSS200, and 1stExon as promoter regions.

### Developing a 5mC score to quantify 5mC clusters

We comprehensively developed a 5mC score to quantify the 5mC subtype of individual patients. The process to establish the 5mC score was similar to that in previous studies [36–44]. First, univariate Cox analysis was performed on the above DEGs (5mC gene signature) to identify prognostic DEGs. Then, principal component analysis was further performed on those prognostic DEGs to calculate principal component 1, which was used for 5mC score calculation in this study.

$$ 5\mathrm{mC}\ \mathrm{score}=\sum \mathrm{PC}{1}_i $$

*i* shows the expression of 5mC cluster-related prognostic DEGs.

For all public BLCA cohorts, we calculated the 5mC score based on the prognostic DEGs. For the Xiangya cohort, we calculated the 5mC score directly based on the 5mC gene signature because the survival data of this cohort were not available. Similarly, we calculated the 5mC score directly based on the 5mC gene signature to ensure the comparability of results in the pancancer analyses.

### Depicting the classical molecular subtypes of BLCA

In this study, we analyzed seven different classical molecular systems, including CIT, Lund, MDA, TCGA, Baylor, UNC, and Consensus subtypes, by using the ConsensusMIBC and BLCAsubtyping R packages based on the RNA expression matrix [8–14]. Despite the presence of numerous subtypes in these systems, BLCA can be generally divided into luminal and basal subtypes. We evaluated the accuracy of the 5mC score in predicting classical molecular subtypes by using receiver operator curves (ROCs).

### Depicting the immunological characteristics of the TME in BLCA

The TME is a complicated and heterogeneous system composed of various immunomodulators, tumor-infiltrating immune cells (TIICs), and inhibitory immune checkpoints. Other immunological characteristics include cancer immunity cycles and T cell inflamed score (TIS). We described these immunological characteristics and the corresponding algorithms in our previous study [45]. Briefly, we summarized 122 immunomodulators (MHCs, chemokines, immune stimulators, and receptors) [46]. Then, we calculated the infiltration level of TIICs in the TME using seven independent algorithms: Cibersort, Cibersort-ABS, MCP-counter, quanTIseq, TIMER, xCell, and TIP [47–52]. In addition, we summarized the effector genes of several critical TIICs (including CD8+ T cells, natural killer cells (NK), dendritic cells (DCs), macrophages, and Th1 cells) and collected 22 inhibitory immune checkpoints [53]. Finally, we estimated the enrichment scores of several stromal signatures, including EMT1, EMT2, EMT3, and the panfibroblast TGFβ response signature (F-TBRS) [36, 54].

The cancer immunity cycles included seven critical steps: cancer antigen release and presentation (Steps 1 and 2), anticancer immune priming and activation (Step 3), immune cell trafficking (Step 4), immune cell infiltration into the TME (Step 5), T cell recognition of cancers (Step 6), and killing of cancer cells (Step 7) [55]. Then, we calculated the activities of these steps as described previously [52]. The pancancer TIS, which could reflect the pre-existing anticancer immunity and predict the clinical response of ICB, was calculated based on eighteen IFN-γ–responsive genes [56].

$$ \mathrm{TIS}={\sum}_{\upgamma =1}^{18}{\upbeta}_{\upgamma}{\mathrm{X}}_{\upgamma} $$

where *β*_*γ*_ is a weighted coefficient predefined in the previous study, and *X*_*γ*_ is the *γ*th gene’s expression level (Table S1I). Furthermore, we previously collected ten genes that could predict the risk of ICB-associated hyperprogression [45].

### The difference of regulon expression and gene fusion events among 5mC clusters

To further depict the molecular differences between 5mC clusters, we analyzed 23 BLCA associated “regulator” gene expression profiles (regulons) between 5mC clusters [8, 14]. The list of 23 regulons is provided in Table S1J. In addition, we collected the gene fusion data from Robertson’s study [8]. Gene fusion events, which occurred at least on two samples, were included in our analysis.

### Collecting classical molecular subtype-specific signatures, therapeutic-specific signatures, and other functional pathways

The Bladder Cancer Molecular Taxonomy Group summarized twelve molecular subtype-specific signatures [14]. Critical therapeutic-specific signatures, including several oncogenic pathways that shaped a noninflamed TME, signatures related to targeted therapy, and signatures related to radiotherapy, were summarized previously [45]. Additionally, the mutation status of several critical genes, including RB1, ATM, ERBB2, ERCC2, and FANCC, predicted the response to neoadjuvant chemotherapy in BLCA. Mariathasan et al. identified nineteen gene signatures related to the clinical response to an anti-PD-L1 agent (atezolizumab) in BLCA [54]. The enrichment scores of these signatures in BLCA samples were calculated using the ssGSEA algorithm [57]. Then, we determined the role of the 5mC subtype and 5mC score in predicting the sensitivities of these therapies. Furthermore, we extracted BLCA-related drugs and drug-target genes from the DrugBank database to further analyze the role of the 5mC score and 5mC subtype in predicting therapeutic opportunities [58]. In addition, we collected 50 hallmark biological pathways, 189 oncogenic pathways, and 186 KEGG pathways from the MSigDB database [59].

### Statistical analysis

We applied the Pearson and Spearman coefficients to analyze the correlations between continuous variables. We used the *t*-test to compare the differences in continuous variables between two groups if the continuous variables fit the normal distribution. Otherwise, we applied the Mann-Whitney *U* test. We used chi-square and Fisher’s exact tests to analyze the difference between categorical variables. For the 5mC score, we applied the “survcutpoint” function to determine the optimal cutoffs. Then, samples were classified into high and low 5mC score groups based on the cutoff. The Kaplan-Meier method was used to plot the survival curves, while the log-rank test was applied to calculate the statistical significance. All statistical analyses were conducted using R software, version 3.6.3 (http://www.r-project.org). The level of significance was set at *P* < 0.05, and all statistical tests were two-sided.

## Results

### Landscape and multiomics analysis of 5mC regulators in BLCA

5mC is a dynamic and reversible process mediated by several distinct regulators that plays critical roles in various biological processes in cancers (Fig. S1A). As shown in Fig. S1B, the 21 5mC regulators were significantly correlated with each other. In the TCGA-BLCA cohort, most of the 5mC regulators were significantly highly expressed in bladder cancer tissues compared with normal tissues (Fig. S1C). Furthermore, this imbalance in the expression of 5mC regulators between bladder cancer tissues and normal tissues was also observed in the Xiangya cohort (Fig. S1D). More importantly, we confirmed the cancer-specific overexpression patterns of 5mC regulators from the aspect of the single-cell RNA sequence. In the Xiangya scRNA set, after quality control, 6798 qualified single cells were visualized with 2D tSNE and classified into seven cell types, including cancer cell, T cell, iCAF, mCAF, myloid, B cell, and endothelial (Fig. S2). A majority of 5mC regulators were expressed explicitly on cancer cells, such as DNMT3A, MBD1, MBD3, UNG, NEIL1, ZBTB33, NTHL1, SMUG1, TDG, UHRF1, TET1, TET2, and TET3 (Fig. S3). Six 5mC regulators, including DNMT1, MECP2, MBD2, ZBTB38, MBD4, and UHRF1, were expressed in both cancer and nonmalignant cells. Nonetheless, the proportion of 5mC regulators positively expressed cells was the highest in the type of cancer cells. Similar results were observed in the GSE145137. For instance, several 5mC regulators, including MBD1, MBD2, MDB3, UNG, ZBTB2, UHRF1, UHRF2, and TET3, were only expressed on cancer cells (Fig. S4). For several 5mC regulators which are expressed in both cancer and nonmalignant cells, the proportion of positively expressed cells was the highest in the type of cancer cells. Meanwhile, the majority of 5mC regulators were adverse prognostic factors in the E-MTAB-4321 cohort (Fig. S1E). However, CNV alterations and mutations of 5mC regulators were not frequent in BLCA (Fig. S1F-G). In summary, these results indicated that 5mC regulators may be potential diagnostic and prognostic predictors in BLCA.

### Depicting the 5mC clusters

**Fig. 1:**
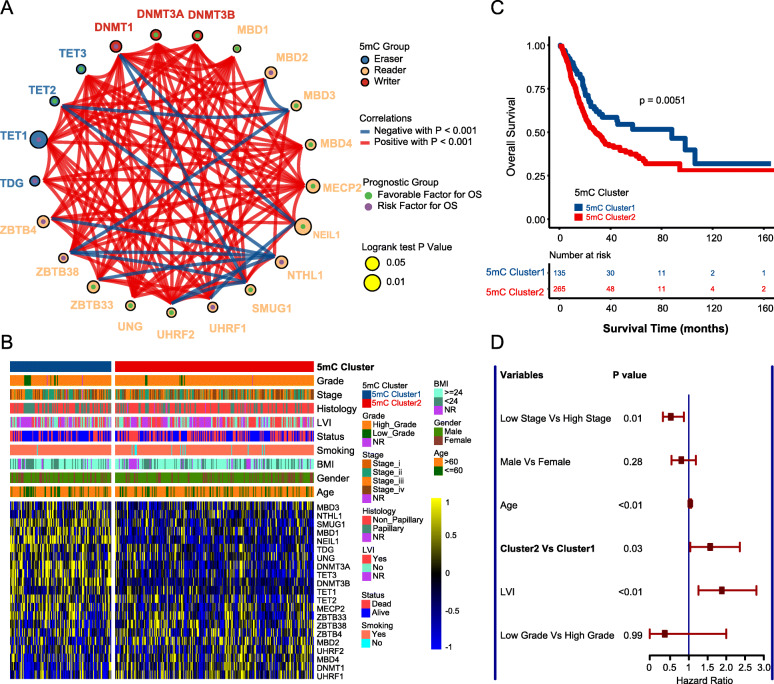
The clinical relevance of 5mC clusters in the TCGA-BLCA cohort. **A** The interactions between 5mC regulators and their prognostic value. The circle size indicates the *p* value of the log-rank test, and the lines linking the 5mC regulators indicate their interactions. **B** The heatmap shows the correlations between 5mC clusters, clinicopathological characters, and 21 5mC regulators’ expression. **C** Survival analysis of 5mC clusters. **D** The forest plot shows the results of multivariable Cox analysis

The workflow of developing 5mC clusters and the 5mC score is shown in Fig. S5A. Figure 1A displays the comprehensive landscapes of 5mC regulators with respect to the prognostic value, correlations, and groups in the TCGA-BLCA cohort. As shown in Fig. S5B-I, the TCGA-BLCA cohort was classified into several clusters based on the mRNA expression of 21 5mC regulators. Notably, only when the cohort was divided into two clusters was the clustering algorithm superior, and different clusters had significant prognostic value. Therefore, 135 patients were classified into 5mC cluster 1, and the rest were classified into 5mC cluster 2. Figure 1B shows the correlations between 5mC clusters and clinicopathological characteristics. Obviously, the 5mC regulators were differentially expressed between the two 5mC clusters. Patients in 5mC cluster 2 had a poorer prognosis than patients in 5mC cluster 1 (Fig. 1C). Further multivariable Cox analysis indicated that the 5mC cluster was an independent prognostic factor after adjusting for stage, grade, age, and LVI (Fig. 1D).

### Developing the 5mC gene signature, 5mC score, and their functional analyses

Figure S6A displays the 5mC score algorithm. First, we identified 401 DEGs (5mC gene signature) between two 5mC clusters (Fig. S6B, Table S2). Then, we highlighted DEGs with the most significant expression differences (|log_2_FC| greater than 2.5) in the volcano map (Fig. S6C). Interestingly, a majority of these highlighted DEGs were BLCA molecular subtype-specific markers. KRT6A, KRT6B, KRT6C, KRT5, KRT14, SERPINB3, SERPINB13, SERPINB4, and DSG3 were basal subtype-specific markers. In contrast, UPK1A, UPK2, UPK3A, KRT20, and SNX31 were luminal subtype-specific markers (Fig. S6C) [60]. This indicated that 5mC clusters may be similar to classical BLCA molecular subtypes. The functions of the 5mC gene signature were obviously enriched in immune-related processes. For example, biological processes (BPs) mainly included leukocyte chemotaxis and myeloid leukocyte migration (Fig. S6D, Table S3A). The molecular functions (MFs) mainly included chemokine/cytokine receptor binding and chemokine/cytokine activity (Fig. S6D, Table S3A). The cellular components (CC) mainly included the MHC protein complex and keratin filament (Fig. S6D, Table S3A). Furthermore, the most significant KEGG pathways of the 5mC gene signature were cytokine-cytokine receptor interactions and chemokine signaling pathways (Fig. S6E, Table S3B). These data suggested that 5mC clusters (5mC gene signature) may play critical roles in modulating TME immunity and regulating the anticancer immune response in BLCA.

Within the 5mC gene signature, 88 DEGs had prognostic value (Table S2). Then, we performed PCA based on these prognostic DEGs to calculate the 5mC score. Eventually, the TCGA-BLCA cohort was divided into a high 5mC score group (*n* = 197) and a low 5mC score group (*n* = 203) based on an optimal cutoff value of the 5mC score. Patients in the high score group had a better prognosis than patients in the low score group (Fig. S6F). As expected, the 5mC score could effectively quantify the 5mC clusters. 5mC cluster 1 belonged to the high 5mC score group, and the low 5mC score group belonged to 5mC cluster 2 (Fig. S6G-H).

**Fig. 2:**
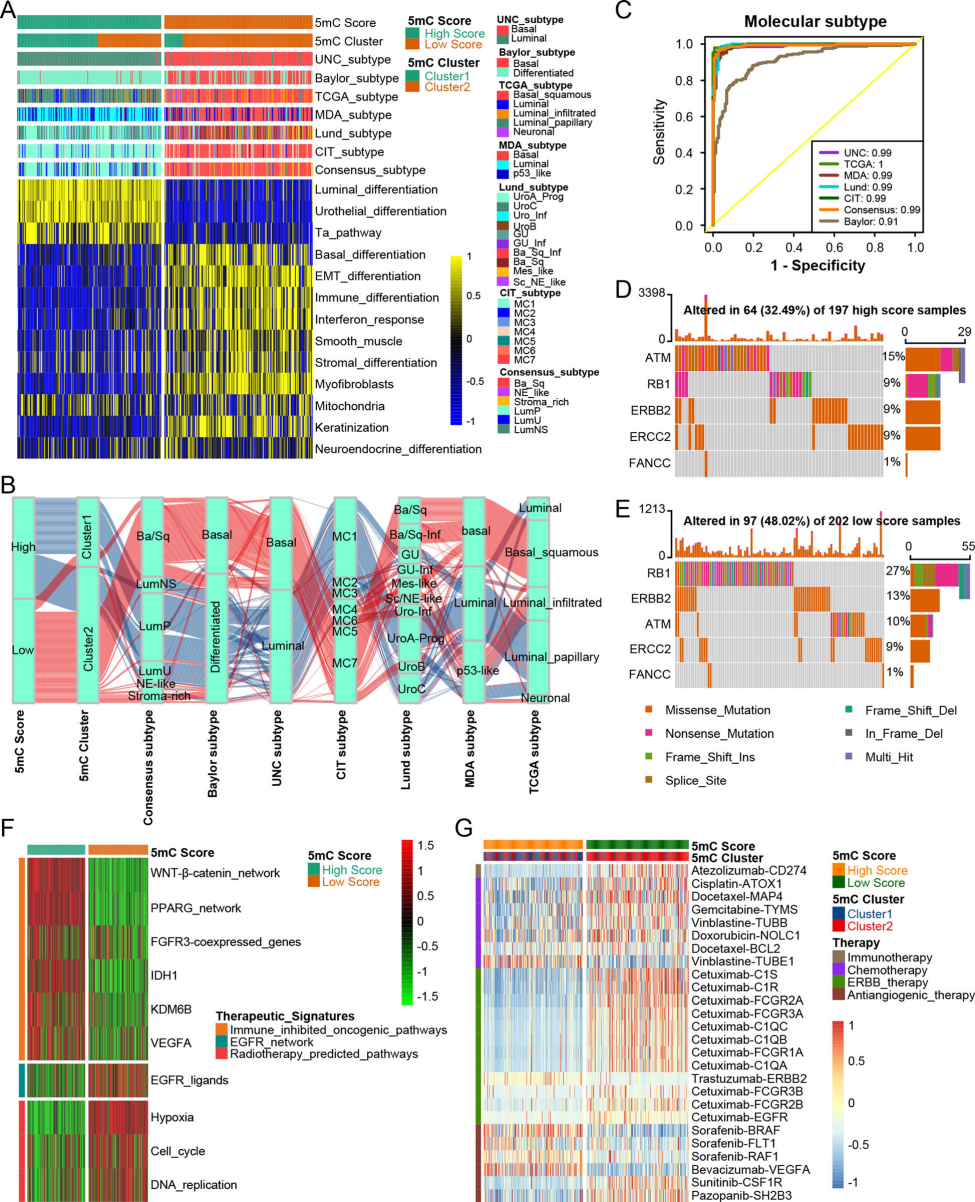
5mC clusters accurately predicted classical molecular subtypes and therapeutic opportunities in the TCGA-BLCA cohort*.* **A**, **B** The correlations between 5mC clusters, 5mC score, and seven classical molecular subtype classifications. **C** ROC curves showed the accuracy of the 5mC score in predicting classical molecular subtypes. **D**, **E** The overall mutation rates of neoadjuvant chemotherapy-related genes in the 5mC score groups. **F** The correlations between the 5mC score and the enrichment scores of several therapeutic signatures, such as targeted therapy and radiotherapy. **G** The correlations between the 5mC score and the BLCA-related drug-target genes were screened from the DrugBank database

Next, we compared the differences in the mutation profiles, hallmark pathways, oncogenic pathways, and KEGG pathways between the 5mC score groups. A majority of hallmark pathways were differentially enriched between the two 5mC score groups (Fig. S7A). Most of the metabolic hallmark signatures were enriched in the high 5mC score group. In contrast, most proliferation-related hallmark signatures, DNA damage-related pathways, development-related pathways, and cellular component-related pathways were significantly enriched in the low 5mC score group. Meanwhile, all immune-related hallmark pathways, such as interferon-alpha/gamma response, were significantly enriched in the low 5mC score group. The results from the PURE-01 study demonstrated that the activation of the interferon-alpha/gamma response predicted a higher ICB response rate in BLCA [61]. Similarly, most of the KEGG pathways were differentially enriched between the two 5mC score groups (Fig. S8). In particular, the immune-related KEGG pathways were significantly enriched in the low 5mC score group. This indicated that the low 5mC score group (5mC cluster 2) may be more sensitive to ICB. A previous study suggested that TP53 and RB1 mutations induced genomic instability and promoted the pathogenesis of BLCA [62]. In this study, the mutation rates of TP53 (55% vs. 40%) and RB1 (27% vs. 9%) were significantly higher in the low 5mC score group than in the high 5mC score group (Fig. S7B-C, Fig. 2D, E). Meanwhile, a majority of oncogenic pathways were significantly enriched in the low 5mC score group (Fig. S7D). These results may explain why the low 5mC score group (5mC cluster 2) had a poorer prognosis.

Collectively, the 5mC gene signature and 5mC score comprehensively reflect the biological characteristics of BLCA, including TME immunity and prognosis.

### 5mC clusters are effective alternatives to classical molecular subtypes and accurately predict therapeutic opportunities in BLCA

Figure 2A, B displays the correlations between 5mC clusters, 5mC score groups, and seven classical molecular subtype classifications. The high 5mC score group and 5mC cluster 1 represented the luminal subtype, which was characterized by luminal differentiation, urothelial differentiation, and the Ta pathway. Conversely, the low 5mC score group (5mC cluster 2) indicated the basal subtype, which was characterized by basal differentiation, EMT differentiation, immune differentiation, interferon response, and keratinization.

As shown in Fig. S9A, a majority of BLCA samples belonged to the basal or luminal subtypes regardless of the molecular systems, although some other subgroups (such as the stromal subtype and NE subtype) had a very low proportion. For instance, two molecular systems (Baylor and UNC) only included basal and luminal subtypes. In the TCGA molecular system, only 4% of samples were NE subtype. Similarly, in the consensus molecular system, only 10% of samples were classified into other subtypes (2% NE and 8% stromal subtypes). These results suggested that the basal and luminal subtypes may reflect the molecular characteristics of most BLCA patients.

The ROC curves showed that the 5mC score predicted classical molecular subtypes with high accuracy ranging from 0.91 to 1 (Fig. 2C). These results were successfully validated in several independent external cohorts (Fig. S10A-D). Therefore, we believed that the binary 5mC cluster systems could also reflect the molecular characteristics of most BLCA patients. Certainly, there was an inescapable limitation for binary cluster systems to reflect the molecular characteristics of other infrequent subtypes, such as NE and stromal subtypes. To further explore the role of the 5mC score in quantificationally distinguishing the different rare subtypes, we compared the difference in 5mC score between basal subtype, luminal subtype, and other subtypes. In line with the results from Fig. 2A, B, basal subtypes had the lowest 5mC score, while luminal subtypes had the highest 5mC score. Interestingly, other rare subtypes (stromal and NE subtypes) had an intermediate score (Fig. S9B). This phenomenon was observed in all molecular subtype systems. Therefore, the 5mC score could make up for the shortcomings of the binary 5mC cluster system to quantitatively reflect the biological characteristics of other rare subtypes.

**Fig. 3:**
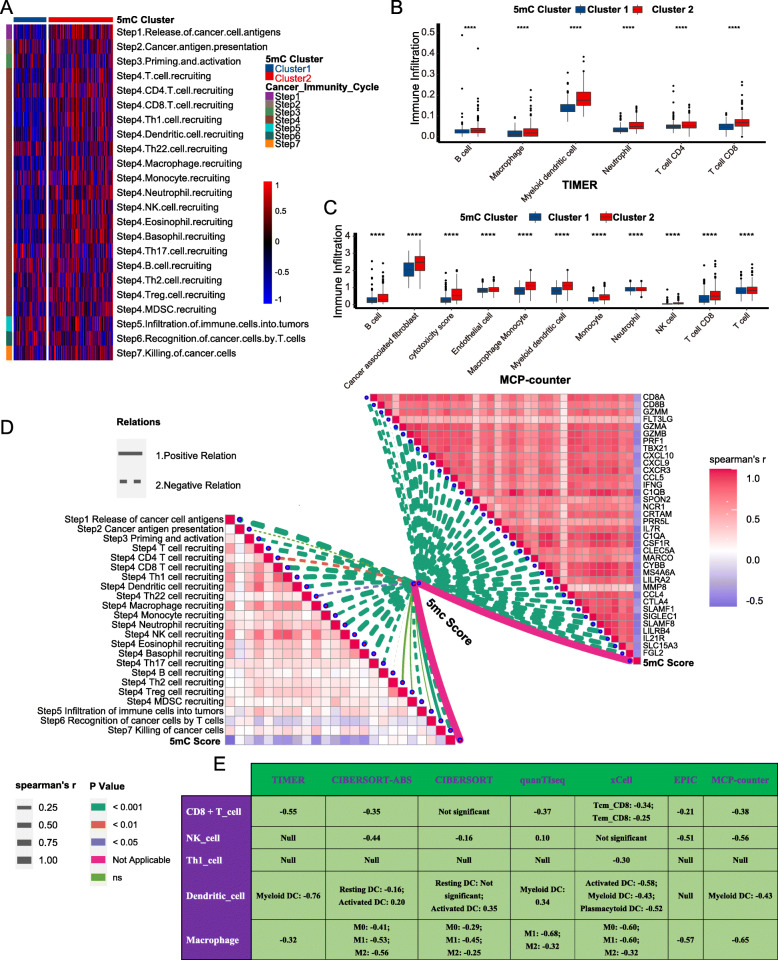
The 5mC clusters and 5mC score correlated with immune phenotypes in the TCGA-BLCA cohort. **A** The differences in cancer immunity cycles between 5mC clusters. **B**, **C** The differences in infiltration levels of TIICs between 5mC clusters in the TIMER and MCP-counter algorithms. The asterisks indicate a statistically significant *p* value calculated using the Mann-Whitney *U* or *t*-test (*P < 0.05; **P < 0.01; ***P < 0.001). **D** The lower left part indicates the correlations between the 5mC score and cancer immunity cycles; the upper right part shows the correlations between the 5mC score and effector genes of several anticancer TIICs, including CD8+ T cells, NK cells, macrophages, Th1 cells, and dendritic cells. **E** The correlation between the 5mC score and the infiltration levels of five anticancer TIICs (CD8+ T cells, NK cells, macrophages, Th1 cells, and dendritic cells), which were calculated using seven independent algorithms.

**Fig. 4:**
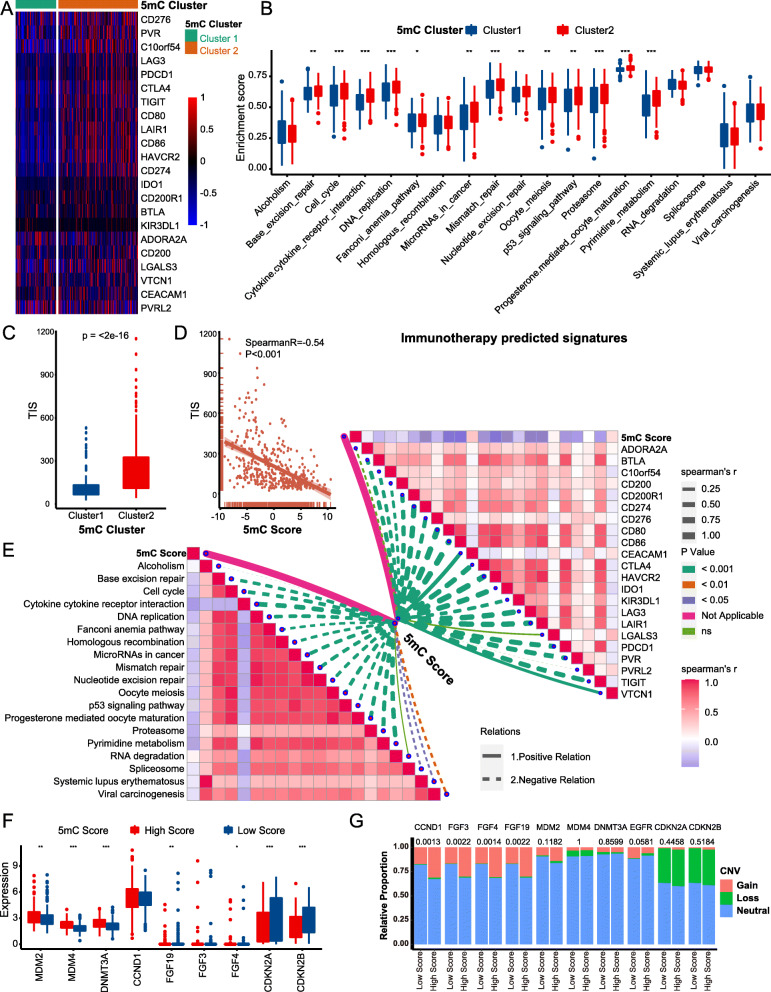
The 5mC clusters and 5mC score correlated with predictors of ICB response in the TCGA-BLCA cohort. **A** The differences in the expression of 22 immune checkpoints between 5mC clusters. **B**, **C** The differences in the enrichment scores of positive ICB response-related signatures and the TIS between 5mC clusters. **D** The correlations between the 5mC score and TIS. **E** The lower left part indicates the correlations between the 5mC score and the enrichment scores of positive ICB response-related signatures; the upper right part shows the correlations between the 5mC score and the expression of 22 immune checkpoints. **F** The difference in mRNA expression of hyperprogression-associated genes between the 5mC score groups. The asterisks indicate a statistically significant *p* value calculated using the Mann-Whitney *U* or *t*-test (*P < 0.05; **P < 0.01; ***P < 0.001). **G** The difference in copy number variation patterns of hyperprogression-associated genes between the 5mC score groups. The *p* value was calculated with the Fisher *t*-test

Several studies demonstrated that tumor purity may influence the molecular subtypes [32, 63]. Fortunately, the purity adjusted 5mC clusters were highly consistent with the original 5mC clusters in this study (Fig. S11A). Only one patient was reclassified into another subtype (Fig. S11B, Table S3C). This highlighted the robustness of our 5mC cluster systems, which the tumor purity may not influence. There were two possible explanations for this result. The first one was that 5mC regulators were specifically overexpressed in cancer cells (Figs. S1C-D, S3, S4). The second one was that the overall tumor purity of the TCGA-BLCA samples was satisfied and acceptable when most samples’ tumor purity (CPE) (84.67%) was higher than 60% (Table S1H). However, the 5mC score was positively related to the purity in five algorithms (Fig. S11C). Patients with a higher 5mC score had higher tumor purity, which indicated lower immune and stromal infiltration in the TME. Consistently, samples with higher 5mC scores represented a luminal subtype with lower immune infiltration and stromal signature enrichment scores (Figs. 2A, 3, 4). The closed correlation between the 5mC score and purity may be due to the fact that the 5mC gene signature contained many immune-related genes. Overall, the tumor purity was more inclined to be regarded as a TME internal character, reflecting the stromal and immune-related features.

The overall mutation rate of neoadjuvant chemotherapy-related genes was significantly higher in the low 5mC score group than in the high 5mC group (48.02% vs. 32.49%) (Fig. 2D, E). This indicated that patients in the low 5mC score group may be more sensitive to neoadjuvant chemotherapy. Meanwhile, patients in the low 5mC score group may be more sensitive to EGFR targeted therapy and radiotherapy (Fig. 2F). In contrast, several immunosuppressive oncogenic pathways were significantly enriched in the high 5mC score group, including the WNT-β-catenin network, PPARG network, FGFR3 network, IDH1, KDM6B, and VEGFA. Therefore, targeting these oncogenic pathways may offer promising therapeutic opportunities for BLCA patients in the high 5mC score group. All of these observations were revalidated in several external BLCA cohorts (Fig. S12A-B). Furthermore, we successfully confirmed the above results in the DrugBank database (Fig. 2G). Patients in the low 5mC score group (5mC cluster 2) were more sensitive to chemotherapy drugs (cisplatin, docetaxel, and gemcitabine), ERBB therapy (cetuximab), and immunotherapy (atezolizumab). However, patients in the high 5mC score group (5mC cluster 1) may be more sensitive to antiangiogenic therapy drugs (sorafenib and bevacizumab).

Collectively, 5mC clusters may be economical and simpler alternatives to classical molecular subtypes. Meanwhile, 5mC clusters and 5mC scores could predict the response to several treatments in BLCA.

### The distinct methylation patterns between 5mC clusters

We firstly screened 49904 DMPs (adj *P* < 0.01) between 5mC clusters (Table S3D). Among these, 142 5mC cluster-specific DMPs were defined (Table S3E). Interestingly, almost all of these DMPs (141/142) were 5mC cluster 2 specific. Only one DMP (cg23507945) was 5mC cluster 1 specific. Meanwhile, the 5mC score was negatively related to the methylation levels of these cluster-specific DMPs (Table S3F). Overall, there was a significantly distinct methylation pattern between 5mC clusters (Fig. S13A). In addition, we explored the associations between the 5mC score and the promoter methylation levels of certain critical cancer-associated genes, such as oncogenes and driver genes . Similarly, the 5mC score was negatively related to most promoter methylation levels of those genes (Table S3G-K). For example, among 1268 promoter methylation probes of oncogenes, 667 probes were negatively related to the 5mC score, while only 94 probes were positively related to the 5mC score. These data further confirmed a higher methylation status in 5mC cluster 2 compared to 5mC cluster 1. Based on these 5mC cluster-specific DMPs, we identified 130 5mC cluster-specific DMGs (Table S3E). Results of GO and KEGG analyses based on 130 5mC cluster-specific DMGs were shown in Table S3L. Among these enriched pathways, 11 GO pathways and 1 KEGG pathway were immune-related (Fig. S13B-C), which suggested that 5mC cluster 2 may be an immune infiltrated phenotype.

Robertson et al. identified several methylation subtypes based on the BLCA-specific hypermethylated or hypomethylated probes [8]. Though these methylation subtypes were related to different clinicopathological features, there was no significant difference in prognosis between these subtypes. Here, we identified 592 BLCA-specific hypermethylated probes and 465 BLCA-specific hypomethylated probes using more stringent criteria (see “Methods” part) (Table S3M-N). We then performed unsupervised clustering based on both BLCA-specific hypermethylated and hypomethylated probes. Unfortunately, the BLCA-specific DMPs clusters were not related to prognosis (Fig. S14). Moreover, there was no association between the BLCA-specific DMPs clusters and the 5mC clusters (Fig. S14). Finally, we performed unsupervised clustering based on 5mC cluster-specific DMPs (Fig. S13D-I). Interestingly, the binary 5mC-specific DMPs clusters were related to prognosis (Fig. S13D). Meanwhile, there was a closed matching relation between the 5mC-specific DMPs clusters and the 5mC clusters (Fig. S13E). 5mC-specific DMPs cluster 1 indicated 5mC cluster 2.

### 5mC clusters (5mC score) predicted immune phenotypes and clinical response of ICB in BLCA

A majority of immunomodulators were downregulated in 5mC cluster 1 (Fig. S15A). Because the activities of cancer immunity cycles are directly determined by the comprehensive performance of immunomodulators, the activities of most cancer immunity cycles were downregulated in 5mC cluster 1, such as the release of cancer cell antigens (Step 1), trafficking of immune cells to tumors (Step 4) (CD8 T cell recruitment, CD4 T cell recruitment, macrophage recruitment, Th1 cell recruitment, NK cell recruitment, DC recruitment), and killing of cancer cells (Step 7) (Figs. 3A, S15C). Consequently, the downregulated activities of these cycles resulted in decreased infiltration levels of corresponding TIICs (including CD8 T cells, CD4 T cells, NK cells, Th1 cells, macrophages, and DCs) in the BLCA TME (Fig. 3B, C, S15D-E). These findings suggested that 5mC cluster 1 may be a noninflamed phenotype. We further analyzed the correlations between 5mC clusters and ICB response predictors. First, most of the immune checkpoints, such as PD-L1, PD-1, and CTLA-4, were downregulated in 5mC cluster 1 (Fig. 4A). Second, the enrichment scores of positive ICB response-related signatures and the TIS were significantly lower in 5mC cluster 1 than in 5mC cluster 2 (Fig. 4B, C). Therefore, 5mC cluster 1 may not be sensitive to ICB.

The 5mC score was negatively related to anticancer immunity in the BLCA TME. Most immunomodulators were downregulated in the high 5mC score group (Fig. S15B). Consistently, the 5mC score negatively correlated with the activities of most cancer immunity cycles (Fig. 3D, Table S4A). As a result, the 5mC score negatively correlated with many anticancer TIICs (including CD8 T cells, CD4 T cells, NK cells, Th1 cells, macrophages, and DCs) and their effector genes, which were cross validated in seven independent algorithms (Fig. 3D, E, Table S4B-C). Furthermore, there were significant adverse correlations between the 5mC score and TIS, enrichment scores of positive ICB response-related signatures, and immune checkpoints (Fig. 4D, E, Table S5).

An inflamed TME was infiltrated by more immune cells and stromal cells. Consistently, the enrichment scores of four stromal signatures, including EMT1, EMT2, EMT3, and F-TBRS, were significantly higher in the 5mC cluster 2 (low 5mC score group) (Fig. S16A-B). In addition, the enrichment score of proliferation was also higher in the low 5mC score group (Fig. S16C).

In summary, 5mC cluster 1 and a high 5mC score predicted a noninflamed phenotype and lower ICB response in BLCA, which was successfully confirmed in several external cohorts (Figs. S17, S18). Moreover, the incidence of ICB-associated hyperprogression may be higher in the high 5mC score group. The mRNA expression and copy number amplification rates of genes positively correlated with ICB-associated hyperprogression, including MDM2, MDM4, DNMT3A, CCND1, FGF3, FGF4, and FGF19, were significantly higher in the high 5mC score group (Fig. 4F, G). In contrast, genes negatively correlated with hyperprogression, such as CDKN2A and CDKN2B, were significantly downregulated in the high 5mC score group.

### A distinct gene fusion patterns and regulon expression profiles between 5mC clusters

In TCGA-BLCA cohort, the most common gene fusions included 10 FGFR3-TACC3 fusions, 9 ITGB6-LOC100505984 fusions, 5 AFF1-PTPN13 fusions, 4 PPARG-SYN2 fusions, 4 GPR110-TNFRSF21 fusions, and 4 TSEN2-PPARG fusions (Fig. S16D). Notably, the FGFR3-TACC3 fusions and AFF1-PTPN13 fusions mainly occurred in the high score group, while ITGB6-LOC100505984 fusions mainly occurred in the low score group. In addition, 7 of 8 PPARG associated fusions occurred in the high score group. In addition, we observed a distinct regulon expression pattern across 5mC clusters. As for 11 luminal subtype-specific regulons, such as RARG, FGFR3, and ERBB2, they were highly expressed in the high 5mC score group (Fig. S16E). This result was similar to the hypothesis that GATA3, FOXA1, and PPARG lead to luminal cell biology for BLCA [64]. In contrast, the expression of 12 basal subtype-specific regulons was significantly higher in the low 5mC score group. Collectively, the distinct gene fusion patterns and regulon expression profiles between 5mC clusters may drive the differences in biological phenotypes between 5mC clusters.

### Validating the role of the 5mC score in stratifying immune phenotypes and clinical response to ICB in a BLCA immunotherapy cohort (IMvigor210)

In the IMvigor210 cohort, patients with higher 5mC scores had better prognoses (Fig. S19A). Patients were divided into several subgroups based on PD-L1 expression on immune cells (IC0, IC1, and IC2+ subgroups) or tumor cells (TC0, TC1, and TC2+ subgroups) and the infiltration status of CD8 T cells in the TME (deserted, excluded, and inflamed subgroups) [54]. Obviously, the 5mC score was the highest in the IC0 (immune cells with the lowest PD-L1 expression) and TC0 (tumor cells with the lowest PD-L1 expression) subgroups and deserted phenotypes (Fig. S19B-D). Additionally, the 5mC score was negatively related to TIS and most of the immune checkpoints, such as PD-L1, PD-1, CTLA-4, and TIM-3 (Fig. S19E-F). Meanwhile, the effector genes of several anticancer TIICs were significantly downregulated in the high 5mC score group (Fig. S19G). These results confirmed that the high 5mC score group represented a noninflamed phenotype.

Next, we analyzed the correlations between the 5mC score and ICB response in three different immune phenotype subgroups. As expected, in the deserted phenotype subgroup, the ICB response rate in the high 5mC score group was significantly lower than that in the low 5mC score group (Fig. S19H). This result indicated that the high 5mC score group represented a noninflamed phenotype. Naturally, the prognosis of patients in the high 5mC score group was poorer due to a lower ICB response rate (Fig. S19I). Interestingly, we observed opposite results in the excluded and inflamed phenotype subgroups. In these two subgroups, the ICB response rates in the high 5mC score group were higher than those in the low 5mC score group (Fig. S19J, L). Certainly, the prognosis of patients in the high 5mC score group in these subgroups was better due to higher ICB response rates (Fig. S19K, M). Such opposite results could be explained by the comprehensive cross-talk between the 5mC score and other ICB response determinants, such as the panfibroblast TGFβ response signature (F-TBRS). F-TBRS attenuated the clinical response to PD-L1 blockade by contributing to T cell exclusion in BLCA [54]. Previous results from the IMvigor210 cohort indicated that the enrichment score of F-TBRS was the lowest in the deserted phenotype subgroup compared with that in the excluded or inflamed phenotype subgroup. In our study, the 5mC score was the highest in the deserted phenotype subgroup (Fig. S19D). Therefore, the ICB response in the deserted phenotype subgroup may be mainly determined by the 5mC score rather than F-TBRS. Conversely, the 5mC score was obviously lower in the excluded and inflamed phenotype subgroups, but the enrichment score of F-TBRS was significantly higher. Thus, the ICB response in these two subgroups may be determined by other factors, such as F-TBRS, instead of the 5mC score. Of course, further research is needed to demonstrate the importance of interactions between the 5mC score and F-TBRS in determining the clinical response to ICB.

### Validating the roles of the 5mC score in the Xiangya cohort

**Fig. 5:**
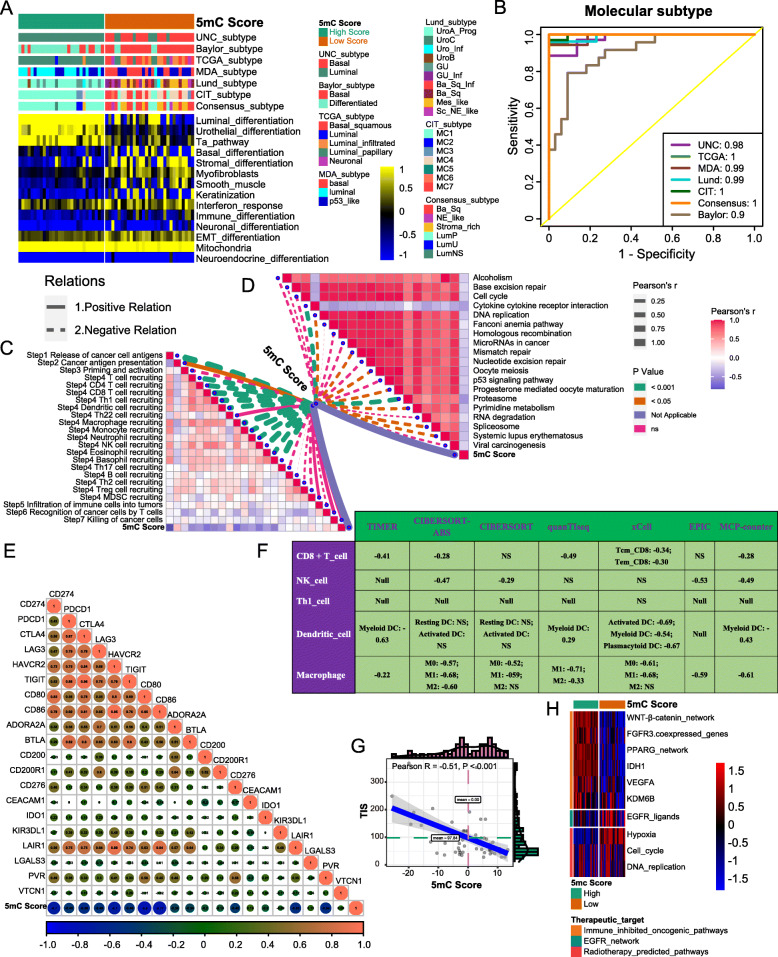
Validating the roles of the 5mC score in the Xiangya cohort. **A** The correlations between the 5mC score and seven classical molecular subtype classifications. (B) ROC curves showed the accuracy of the 5mC score in predicting classical molecular subtypes. **C** The correlations between the 5mC score and the cancer immunity cycles. **D** The correlations between the 5mC score and the enrichment scores of positive ICB response-related signatures. **E** The correlations between the 5mC score and 22 immune checkpoints. **F** The correlation between the 5mC score and the infiltration levels of five anticancer TIICs (CD8+ T cells, NK cells, macrophages, Th1 cells, and dendritic cells), which were calculated using seven independent algorithms. **G** The correlations between the 5mC score and TIS. **H** The differences in the enrichment scores of several therapeutic signatures, such as targeted therapy and radiotherapy, between the 5mC score groups

In our own cohort (Xiangya cohort), we found that the 5mC score could accurately predict classical molecular subtypes (Fig. 5A). The AUC ranged from 0.99 to 1, except in the Baylor subtype system (AUC = 0.9) (Fig. 5B). In addition, the 5mC score was negatively correlated with the activities of many anticancer immunity steps (Fig. 5C, Table S6A). Subsequently, the 5mC score was also negatively related to the infiltration levels of CD8 T cells, NK cells, Th1 cells, DCs, and macrophages in seven independent algorithms (Fig. 5F, Table S6B). Meanwhile, there were significantly negative correlations between the 5mC score and immune checkpoints, TIS, and enrichment scores of positive ICB response-related signatures (Fig. 5D, E, G, Table S6C). These data demonstrated that the 5mC score could effectively stratify the immune phenotypes of BLCA. In addition, the 5mC score was able to predict the clinical response to other treatments, including EGFR targeted therapy, radiotherapy, and several therapies targeting immune-inhibited oncogenic pathways (Fig. 5H).

### Pancancer analyses of the 5mC score

We further evaluated the role of the 5mC score across cancers. Notably, the 5mC score was related to prognosis in many cancers, such as thymoma, lower-grade glioma, and kidney renal clear cell carcinoma (Fig. S20A, Table S7B). In addition, the 5mC score was negatively correlated with the expression of four critical immune checkpoints, PD-L1, PD-1, CTLA-4, and LAG-3, in most cancers (Fig. S20B-E, Table S7C-F). Aberrant DNA methylation may influence cancer immunogenicity, such as TMB and MSI [65]. Here, we revealed that the 5mC score was related to the TMB and MSI in many cancers (Fig. S20F-G, Table S7G-H). Moreover, the 5mC score was significantly related to the stemness indices of many cancers, such as testicular germ cell tumors and lung squamous cell carcinoma (Fig. S21, Table S7I-N). Therefore, the 5mC score reflected many biological characteristics of the TME, such as anticancer immunity, immunogenicity, and cancer stemness, in pancancer analyses. It may be a generalizable predictor of prognosis and ICB response across cancers.

### The 5mC score was a valuable predictor of the response to immunotherapy in multiple immunotherapy cohorts

**Fig. 6:**
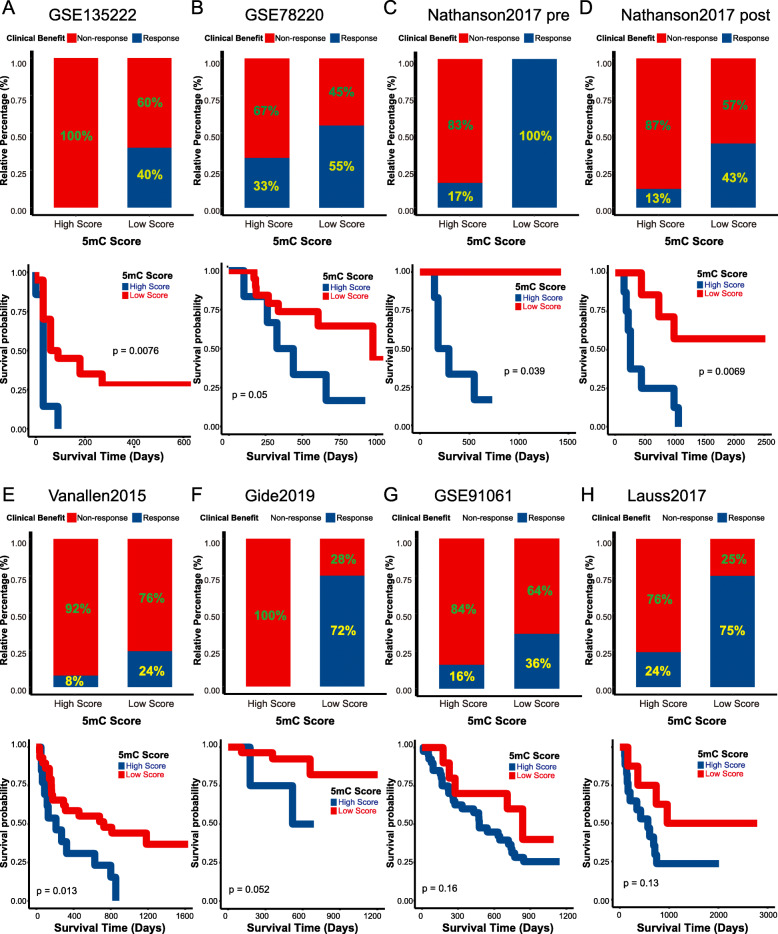
The 5mC score predicted the response to immunotherapy in several immunotherapy cohorts. **A** Non-small-cell lung cancer (GSE135222 cohort): The upper part indicates the proportion of patients who responded to ICB in the low and high 5mC score groups; the lower part shows the survival analysis of the 5mC score groups. **B–H** Melanoma (Six ICB cohorts: GSE78220, Nathanson2017 pre, Nathanson2017 post, VanAllen2015, Gide2019, and GSE91061 cohorts; One adoptive T cell therapy cohort: Lauss2017 cohort): Upper part indicated the proportion of patients with responses to immunotherapy in low and high 5mC score groups; lower part shows the survival analysis of 5mC score groups

Here, we explored the role of the 5mC score in predicting the ICB response in other cancers (including melanoma, non-small cell lung cancer, and gastric cancer) from nine immunotherapy-related cohorts (eight ICB cohorts and one adoptive T cell therapy cohort). First, we found that the 5mC score was negatively correlated with most immune checkpoints in eight ICB cohorts (Figs. S22, S23, S24A). In line with this, the ICB response rates were obviously lower in the high 5mC score group than in the low 5mC score group (Figs. 6A–G, S24B). The prognosis of the high 5mC score group was also poorer due to lower ICB response rates (Fig. 6A–G). Similar results were observed in the adoptive T cell therapy cohort (Fig. 6H). This evidence reconfirmed that the 5mC score was a valuable predictor of immunotherapy response across cancers.

## Discussion

Depicting TME heterogeneity is necessary to achieve precision medicine for BLCA. Although classical molecular subtypes can effectively reflect TME heterogeneity [4], their clinical application may be limited by several issues, such as the complex sequencing method, high economic burden, and long detection period. Here, we first developed a novel 5mC regulator-mediated molecular subtype system that could accurately predict classical molecular subtypes, immune phenotypes, clinical outcomes, and therapeutic opportunities in BLCA. Furthermore, we developed the 5mC score to quantify an individual’s 5mC subtype.

Several molecular subtype predictors were developed previously, such as BASE47 [11]. However, the value of BASE47 in guiding clinical decision making has not been evaluated. Other simplified molecular subtype systems have been developed based on immunohistochemical markers or histological images [66, 67]. However, the accuracy of these simplified systems should be further improved. Compared to previous systems, the 5mC score can stratify basal and luminal subtypes with high accuracy, ranging from 0.98 to 1, in several independent algorithms. More importantly, the 5mC score can predict the clinical response to several treatment options, including neoadjuvant chemotherapy, targeted therapy, radiotherapy, and ICB. A high 5mC score represented a luminal subtype characterized by high urothelial differentiation. The mutation rate of RB1 was significantly lower in the high 5mC score group, which indicated that the high 5mC score group (5mC cluster 1) may not be sensitive to neoadjuvant chemotherapy. Meanwhile, the high 5mC score group (5mC cluster 1) was not sensitive to ERBB therapy and radiotherapy. Fortunately, several immune-inhibited oncogenic pathways were enriched in the high 5mC score group (5mC cluster 1). Therefore, targeting these pathways may offer promising treatment options for patients in the high 5mC score group. As noted in our previous study, these immune-inhibited oncogenic pathways may lead to a noninflamed TME [45]. In line with this, we found that the high 5mC score group had a noninflamed phenotype characterized by low anticancer immunity. Therefore, targeting these pathways may convert the high 5mC score group (5mC cluster 1) into an inflamed phenotype, which may be sensitive to ICB again. In general, the ability to accurately discriminate distinct molecular subtypes and guide precision medicine may promote the widespread clinical application of the 5mC score in BLCA.

DNA methylation plays critical roles in modifying the anticancer immune response in two main ways: directly affecting tumor cells or directly regulating the differentiation and maturity of TIICs [65, 68–71]. In addition, aberrant DNA methylation may lead to ICB resistance. Hypomethylating agents could enhance the recruitment of anticancer TIICs to reverse ICB resistance by promoting the type I interferon response [72]. In this study, the 5mC clusters and 5mC score reflected many of the immune hallmarks of the BLCA TME. The differentially expressed genes between 5mC clusters were significantly enriched in immune-related pathways, such as leukocyte chemotaxis and cytokine/chemokine signaling pathways. The 5mC score was negatively related to most of the immunomodulators, such as CXCL9, CXCL10, and CXCR3, which were critical for the infiltration of anticancer TIICs. In line with this, the 5mC score was negatively correlated with the activities of several cancer immunity cycles, such as the release of cancer cell antigens, priming and activation, and trafficking of immune cells to tumors. Consequently, the 5mC score negatively correlated with several anticancer TIICs, including CD8 T cells and NK cells. In general, a high 5mC score (5mC cluster 1) indicated a noninflamed phenotype characterized by low pre-existing anticancer immunity. It is well accepted that a noninflamed phenotype is not sensitive to ICB [45]. Consistently, we found that the 5mC score was negatively related to TIS, ICB response positively related signatures, and immune checkpoints. Furthermore, in the IMvigor210 cohort, we confirmed that a high 5mC score was negatively related to the response to ICB. Moreover, a higher 5mC score indicated a higher incidence of ICB-associated hyperprogression. Therefore, the 5mC score was a potential predictor of ICB response in BLCA.

Malta et al. calculated cancer stemness indices based on DNA methylation profiles and mRNA expression matrices to assess the degree of oncogenic dedifferentiation [29]. The stemness indices can reflect TME heterogeneity. Here, we found that the 5mC score was significantly related to the cancer stemness indices in pancancer analyses, which again demonstrated that the 5mC score could reflect TME heterogeneity. Furthermore, Malta et al. revealed that cancer stemness indices were negatively correlated with TIIC infiltration and PD-L1 expression across cancers. Consistently, the 5mC score was negatively related to PD-L1, PD-1, CTLA-4, and LAG-3 in all cancers in our study. These findings suggested that a higher 5mC score may predict a lower ICB response in pancancers, caused by higher cancer stemness indices and lower anticancer immunity. Furthermore, we directly validated the role of the 5mC score in predicting ICB response in nine independent immunotherapy cohorts. As expected, a higher 5mC score predicted a lower ICB response. Therefore, the 5mC score may be a potential generalizable predictor of ICB response across cancers.

Certainly, there were several drawbacks in this study. First, the sample size of the Xiangya cohort should be further enlarged in the future. Second, there were no survival data of the Xiangya cohort because the follow-up period was not sufficient. Third, we did not analyze the differences in the overall DNA methylation profiles, including hyper- or hypomethylated CpG sites, between the 5mC clusters.

## Conclusions

The novel 5mC regulator-based subtype system reflects many aspects of bladder cancer biology and provides new insights into bladder cancer treatment. The 5mC score was capable of quantifying the 5mC subtype, identifying distinct molecular subtypes, and stratifying therapeutic opportunities in BLCA. Meanwhile, the 5mC score may be a generalizable predictor of ICB response and prognosis across cancers.

## Supplementary Information

**Additional file 1: Table S1.** (A) Datasets included in this study for identifying distinct 5mC methylation modification patterns. (B) Abbreviations of cancers in TCGA. (C) Basic information of TCGA-BLCA cohort. (D) Basic information of Xiangya cohort. (E) Basic information of GSE32894 cohort. (F) Basic information of GSE48075. (G) Basic information of E-MTAB-4321. (H) The purity of TCGA-BLCA samples. (I) T cell inflamed score algorithm. (J) The list of BLCA associated regulons. **Table S2.** (A) A total of 401 differentially expressed genes (DEGs) between 5mC subtypes and their prognostic value. **Table S3.** (A) GO analysis of 5mC gene signature. (B) KEGG analysis of 5mC gene signature. (C) The purity adjusted 5mC clusters. (D) Differential analysis of methylation probes between 5mC clusters. (E) The 5mC clusters specific differential methylation probes and genes. (F) The correlations between 5mC score and the 5mC cluster differential methylation probes. (G) Correlations between the 5mC score and the probes of 5mC genes. (H) Correlations between the 5mC score and the probes of Oncogenes. (I) Correlations between the 5mC score and the probes of Tumor suppressor genes. (J) Correlations between the 5mC score and the probes of Driver genes. (K) Correlations between the 5mC score and the probes of Kinase genes. (L) GO and KEGG analysis of the 5mC cluster specific genes. (M) Cancer specific hypermethylation sites. (N) Cancer specific hypomethylation sites. **Table S4.** (A) Correlations between the 5mC score and anticancer immunity cycles. (B) Correlations between the 5mC score and effector genes of several anticancer TIICs. (C) Correlations between the 5mC score and TIICs in six algorithms. **Table S5.** (A) Correlations between the 5mC score and 22 immune checkpoints; (B) Correlations between the 5mC score and enrichment scores of positive ICB response-related signatures. **Table S6.** (A) Correlations between the 5mC score and anticancer immunity cycles in the Xiangya cohort. (B) Correlations between the 5mC score and TIICs in six algorithms in the Xiangya cohort. (C) Correlations between the 5mC score and ICB response-related signatures in the Xiangya cohort. **Table S7.** (A) The calculated 5mC score in pancancers. (B) The prognostic analyses of the 5mC score across cancers using a univariate Cox regression model. (C) Correlations between the 5mC score and PD-L1 across cancers. (D) Correlations between the 5mC score and PD-1 in all cancers. (E) Correlations between the 5mC score and CTLA-4 across cancers. (F) Correlations between the 5mC score and LAG-3 in all cancers. (G) Correlations between the 5mC score and TMB in all cancers. (H) Correlations between the 5mC score and MSI in pancancers. (I) Correlations between the 5mC score and mRNAsi across cancers. (J) Correlations between 5mC score and EREG.mRNA in pancancers. (K) Correlations between the 5mC score and mDNAsi in pancancers. (L) Correlations between the 5mC score and EREG mDNA across cancers. (M) Correlations between the 5mC score and DMPsi in pancancers. (N) Correlations between the 5mC score and ENHsi in all cancers.

**Additional file 2: Fig. S1.** Landscape and multiomic analysis of 21 5mC regulators in BLCA. **Fig. S2.** Single cell RNAseq analyses. **Fig. S3.** Expression of 21 5mC regulators for all cell types in Xiangya scRNA set. **Fig. S4.** Expression of 21 5mC regulators for all cell types in GSE145137. **Fig. S5.** Overview of the study design and the 5mC molecular subtypes using unsupervised clustering analysis. **Fig. S6.** Developing the 5mC gene signature, 5mC score and their functional analyses in the TCGA-BLCA cohort. **Fig. S7.** Functional analyses of 5mC score groups. **Fig. S8.** The differences in KEGG pathways between the 5mC score groups. **Fig. S9.** (A) The proportions of every subgroup in different molecular subtype systems. (B) The distribution of 5mC score among different subgroups in all molecular subtype systems. **Fig. S10.** The 5mC score accurately predicted classical molecular subtypes in two external validation BLCA cohorts. **Fig. S11.** (A-B) The associations between original 5mC clusters and purity adjusted 5mC clusters. (C) The correlations between 5mC score and five kinds of tumor purities. **Fig. S12.** The 5mC score predicted therapeutic opportunities in two external validation BLCA cohorts. **Fig. S13.** The methylation patterns between the 5mC clusters. **Fig. S14.** The prognostic difference between BLCA-specific DMPs based clusters of the TCGA-BLCA cohort. **Fig. S15.** Correlations between the 5mC subtype, 5mC score and immunological characteristics in the TCGA-BLCA cohort. **Fig. S16.** (A-B) The differences in four stromal signature enrichment scores between 5mC clusters and 5mC score groups.(C) The differences in proliferation signature enrichment score between 5mC score groups. (D) The distributions of gene fusion events between 5mC score groups. (E) The differences in 23 regulons expression between 5mC score groups. **Fig. S17.** The 5mC score correlated with immune phenotypes and ICB response in the GEO BLCA meta-cohort (GSE48075, GSE32894). **Fig. S18.** The 5mC score correlated with immune phenotypes and ICB response in the E-MTAB-4321 cohort. **Fig. S19.** 5mC score stratified immune phenotypes and clinical response of ICB in the IMvigor210 cohort. **Fig. S20.** Pancancer analyses of the 5mC gene signature (5mC score). **Fig. S21.** Correlations between the 5mC gene signature (5mC score) and cancer stemness indices across cancers. **Fig. S22.** Correlations between the 5mC score and 22 immune checkpoints in four immunotherapy cohorts. **Fig. S23.** Correlations between the 5mC score and 22 immune checkpoints in four immunotherapy cohorts. **Fig. S24.** Correlations between the 5mC score and 22 immune checkpoints and ICB response in the Kim 2018 cohort (gastric cancer).

## Data Availability

All data generated from this study are available upon request to the corresponding author.

## Abbreviations

5mC: 5-Methylcytosine
BLCA: Bladder cancer
BP: Biological processes
CC: Cellular component
CNV: Copy number variation
DCs: Dendritic cells
DEGs: Differentially expressed genes
DMGs: Differential methylation genes
DMPs: Differential methylation probes
GEO: Gene Expression Omnibus
GO: Gene Ontology
ICB: Immune checkpoint blockade
KEGG: Kyoto Encyclopedia of Genes and Genomes
MF: Molecular function
MIBC: Muscle-invasive bladder cancer
MSI: Microsatellite instability
NKs: Natural killer cells
ROCs: Receiver operator curves
scRNA: Single-cell RNA
TCGA: The Cancer Genome Atlas
TIICs: Tumor-infiltrating immune cells
TIS: T cell inflamed score
TMB: Tumor mutation burden
TME: The tumor microenvironment
TPM: Transcripts per kilobase million
