# Supplementary material for: 5mC regulator-mediated molecular subtypes depict the hallmarks of the tumor microenvironment and guide precision medicine in bladder cancer
Source: BMC Med. 2021 Nov 26;19:289. doi: 10.1186/s12916-021-02163-6 (PMC8627095; doi:10.1186/s12916-021-02163-6)
Supplement: Supplementary file 2 — Additional file 2: Fig. S1. Landscape and multiomic analysis of 21 5mC regulators in BLCA. Fig. S2. Single cell RNAseq analyses. Fig. S3. Expression of 21 5mC regulators for all cell types in Xiangya scRNA set. Fig. S4. Expression of 21 5mC regulators for all cell types in GSE145137. Fig. S5. Overview of the study design and the 5mC molecular subtypes using unsupervised clustering analysis. Fig. S6. Developing the 5mC gene signature, 5mC score and their functional analyses in the TCGA-BLCA cohort. Fig. S7. Functional analyses of 5mC score groups. Fig. S8. The differences in KEGG pathways between the 5mC score groups. Fig. S9. (A) The proportions of every subgroup in different molecular subtype systems. (B) The distribution of 5mC score among different subgroups in all molecular subtype systems. Fig. S10. The 5mC score accurately predicted classical molecular subtypes in two external validation BLCA cohorts. Fig. S11. (A-B) The associations between original 5mC clusters and purity adjusted 5mC clusters. (C) The correlations between 5mC score and five kinds of tumor purities. Fig. S12. The 5mC score predicted therapeutic opportunities in two external validation BLCA cohorts. Fig. S13. The methylation patterns between the 5mC clusters. Fig. S14. The prognostic difference between BLCA-specific DMPs based clusters of the TCGA-BLCA cohort. Fig. S15. Correlations between the 5mC subtype, 5mC score and immunological characteristics in the TCGA-BLCA cohort. Fig. S16. (A-B) The differences in four stromal signature enrichment scores between 5mC clusters and 5mC score groups.(C) The differences in proliferation signature enrichment score between 5mC score groups. (D) The distributions of gene fusion events between 5mC score groups. (E) The differences in 23 regulons expression between 5mC score groups. Fig. S17. The 5mC score correlated with immune phenotypes and ICB response in the GEO BLCA meta-cohort (GSE48075, GSE32894). Fig. S18. The 5mC score correlated with immune pheno [file 12916_2021_2163_MOESM2_ESM.docx]

**Figure S1. Landscape and multiomic analysis of 21 5mC regulators in BLCA.** (A) The dynamic reversible process of 5mC DNA methylation regulated by 21 5mC regulators (3 "writers," 4 "erasers" and 14 "readers") and their potential biological functions. (B) The correlations between 21 5mC regulators in the TCGA-BLCA cohort. (C-D) The differential expression patterns of 21 5mC regulators in bladder cancer and normal tissues in the TCGA-BLCA cohort and Xiangya cohort, respectively. (E) The prognostic value of 21 5mC regulators in the E-MTAB-4321 cohort. (F) The copy number variation frequency of 21 5mC regulators in the TCGA-BLCA cohort. (G) The mutation profiles of 21 5mC regulators in 399 patients in the TCGA-BLCA cohort.

**Figure S2. Single cell RNAseq analyses.** (A) Copy number variations (CNVs) evaluated per cell by InferCNV. (B) Heatmap of top 10 marker genes of every major cell types. Shown are row z-score. (C-E) The CNV level of different groups and cell types. (F) tSNE plot of single cells defined in this study colored by major cell types.

**Figure S3.** Expression of 21 5mC regulators for all cell types in Xiangya scRNA set.

**Figure S4.** Expression of 21 5mC regulators for all cell types in GSE145137.

**Figure S5. Overview of the study design and the 5mC molecular subtypes using unsupervised clustering analysis.** (A) Overview of study design. (B, D, F, H) Consensus matrices of the TCGA-BLCA cohort for k = 2-5. (C, E, G, I) Survival analysis of different 5mC subtypes of the TCGA-BLCA cohort for k = 2-5.

**Figure S6.** **Developing the 5mC gene signature, 5mC score and their functional analyses in the TCGA-BLCA cohort**. (A) The flow chart of the 5mC score algorithm. (B) The heatmap shows the differentially expressed genes (DEGs) between 5mC subtypes. (C) The volcano plot shows the DEGs, and the DEGs with the most significant expression differences (|log_2_FC| greater than 2.5) are highlighted. (D) Gene Ontology (GO) analysis of the 5mC gene signature. (E) Kyoto Encyclopedia of Genes and Genomes (KEGG) analysis of the 5mC gene signature. (F) Survival analysis of the 5mC score groups. (G) The differences in the 5mC score between the 5mC subtypes. (H) Sankey plot showing the correlations between the 5mC score groups and 5mC subtypes.

**Figure S7. Functional analyses of 5mC score groups.** (A) The differences in hallmark pathways between the 5mC score groups. (B-C) The differences in mutational profiles between 5mC score groups. (D) The differences in oncogenic pathways between the 5mC score groups.

**Figure S8. The differences in KEGG pathways between the 5mC score groups.**

**Figure S9.** (A) The proportions of every subgroup in different molecular subtype systems. (B) The distribution of 5mC score among different subgroups in all molecular subtype systems. The blue marked subgroups were defined as basal subtype, the red marked subgroups were defined as luminal subtype, and the black marked subgroups belonged to other rare subtypes (NE and Stromal subtypes).

**Figure S10. The 5mC score accurately predicted classical molecular subtypes in two external validation BLCA cohorts**. (A) The correlations between the 5mC score and seven classical molecular subtype classifications in the GEO BLCA meta-cohort (GSE48075, GSE32894). (B) ROC curves showed the accuracy of the 5mC score in predicting classical molecular subtypes in the GEO BLCA meta-cohort. (C) The correlations between the 5mC score and seven classical molecular subtype classifications in the E-MTAB-4321 cohort. (D) ROC curves showed the accuracy of the 5mC score in predicting classical molecular subtypes in the E-MTAB-4321 cohort.

**Figure S11.** (A-B) The associations between original 5mC clusters and purity adjusted 5mC clusters. (C) The correlations between 5mC score and five kinds of tumor purities.

**Figure S12. The 5mC score predicted therapeutic opportunities in two external validation BLCA cohorts**. (A) The correlations between the 5mC score and the enrichment scores of several therapeutic signatures, such as targeted therapy and radiotherapy, in the GEO BLCA meta-cohort (GSE48075, GSE32894). (B) The correlations between the 5mC score and the enrichment scores of several therapeutic signatures, such as targeted therapy and radiotherapy, in the E-MTAB-4321 cohort.

**Figure S13**. (A) The 5mC cluster specific differential methylation probes (DMPs) between 5mC clusters. (B-C) The immune related GO and KEGG pathways enriched by the 5mC cluster specific differential methylation genes. (D, F, H) Survival analysis of 5mC cluster specific DMPs based subtypes of the TCGA-BLCA cohort for k = 2-4. (E, G, I) Sankey plot showing the correlations between the 5mC clusters and 5mC cluster specific DMPs based subtypes of the TCGA-BLCA cohort for k = 2-4.

**Figure S14.** The prognostic difference between BLCA specific DMPs based clusters of the TCGA-BLCA cohort (K= 2-5). The Sankey plot showing the correlations between the 5mC clusters and BLCA specific DMPs based clusters of the TCGA-BLCA cohort for k = 2-5.

**Figure S15. Correlations between the 5mC subtype, 5mC score and immunological characteristics in the TCGA-BLCA cohort**. (A-B) The differences in 122 immunomodulators between the 5mC subtype and 5mC score groups. (C) The differences in cancer immunity cycles between 5mC subtypes. (D) The differences in the expression of effector genes of several anticancer TIICs (including CD8+ T cells, NK cells, macrophages, Th1 cells, and dendritic cells) between 5mC subtypes. (E) The differences in the infiltration levels of TIICs calculated by the quanTIseq algorithm between 5mC subtypes.

**Figure S16.** (A-B) The differences in four stromal signature enrichment scores between 5mC clusters and 5mC score groups.(C) The differences in proliferation signature enrichment score between 5mC score groups. (D) The distributions of gene fusion events between 5mC score groups. (E) The differences in 23 regulons expression between 5mC score groups.

**Figure S17.** **The 5mC score correlated with immune phenotypes and ICB response in the GEO BLCA meta-cohort** **(GSE48075, GSE32894).** (A) Survival analysis of 5mC score groups. (B) The differences in 122 immunomodulators between the 5mC score groups. (C) The differences in the expression of effector genes of several anticancer TIICs (including CD8+ T cells, NK cells, macrophages, Th1 cells, and dendritic cells) between the 5mC score groups. (D) The lower left part indicates the correlations between the 5mC score and the enrichment scores of positive ICB response-related signatures; the upper right part shows the correlations between the 5mC score and the expression of 22 immune checkpoints. (E) The correlations between 5mC score and TIS.

**Figure S18.** **The 5mC score correlated with immune phenotypes and ICB response in the E-MTAB-4321 cohort.** (A) Survival analysis of 5mC score groups. (B) The differences in 122 immunomodulators between the 5mC score groups. (C) The differences in the expression of effector genes of several anticancer TIICs (including CD8+ T cells, NK cells, macrophages, Th1 cells, and dendritic cells) between the 5mC score groups. (D) The lower left part indicates the correlations between the 5mC score and the enrichment scores of positive ICB response-related signatures; the upper right part shows the correlations between the 5mC score and the expression of 22 immune checkpoints. (E) The correlations between the 5mC score and TIS.

**Figure S19. 5mC score stratified immune phenotypes and clinical response of ICB in the IMvigor210 cohort.** (A) Survival analysis of 5mC score groups. (B-C) The correlations between the 5mC score and PD-L1 expression on tumor cells and PD-L1 expression on immune cells. (D) The differences in the 5mC score between the three immune phenotypes. The asterisks indicate a statistically significant p-value calculated using the Mann-Whitney U or t-test (*P < 0.05; **P < 0.01; ***P < 0.001). (E) The correlations between the 5mC score and 22 immune checkpoints. (F) The correlations between the 5mC score and TIS. (G) The difference in the effector genes of several anticancer TIICs, including CD8+ T cells, NK cells, macrophages, Th1 cells, macrophages, and dendritic cells, between the 5mC score groups. (H) The proportion of patients responding to ICB in the low and high 5mC score groups in the deserted subgroup. (I) Survival analysis of 5mC score groups in the deserted subgroup. (J, L) The proportion of patients responding to ICB in the low and high 5mC score groups in the excluded subgroup and the inflamed subgroup. (K, M) Survival analysis of 5mC score groups in the excluded subgroup and the inflamed subgroup.

**Figure S20. Pancancer analyses of the 5mC gene signature (5mC score).** (A) The prognostic analyses of the 5mC score across cancers using a univariate Cox regression model. A hazard ratio >1 indicated a risk factor, and a hazard ratio <1 represented a protective factor. (B-E) The correlations between the 5mC score and four immune checkpoints, PD-L1, PD-1, CTLA-4, and LAG-3. The dots represent cancer types. The Y-axis represents the Pearson correlation, while the X-axis represents -log_10_P. (F) The correlations between the 5mC score and TMB in all cancers. (G) The correlations between the 5mC score and MSI across cancers. The asterisks indicate a significant statistical p-value calculated with Spearman correlation analysis. (*P < 0.05; **P < 0.01; ***P < 0.001).

**Figure S21. Correlations between the 5mC gene signature (5mC score) and cancer stemness indices across cancers.** Cancer stemness indices included mRNAsi, EREG.mRNA, mDNAsi, EREG.mDNA, DMPsi, and ENHsi.

**Figure S22. Correlations between the 5mC score and 22 immune checkpoints in four immunotherapy cohorts.** (A) GSE135222 cohort. (B) GSE78220 cohort. (C) Nathanson2017 pre cohort. (D) Nathanson2017 post cohort.

**Figure S23. Correlations between the 5mC score and 22 immune checkpoints in four immunotherapy cohorts.** (A) VanAllen2015 cohort. (B) Gide2019 cohort. (C) GSE91061 cohort. (D) Lauss2017 cohort.

**Figure S24.** **Correlations between the 5mC score and 22 immune checkpoints and ICB response in the Kim 2018 cohort** **(gastric cancer).**
